# Elongator and its epigenetic role in plant development and responses to abiotic and biotic stresses

**DOI:** 10.3389/fpls.2015.00296

**Published:** 2015-04-29

**Authors:** Yezhang Ding, Zhonglin Mou

**Affiliations:** Department of Microbiology and Cell Science, University of FloridaGainesville, FL, USA

**Keywords:** Elongator, tRNA modification, histone acetylation, DNA methylation, plant development, abiotic stress, plant immunity

## Abstract

Elongator, a six-subunit protein complex, was initially isolated as an interactor of hyperphosphorylated RNA polymerase II in yeast, and was subsequently identified in animals and plants. Elongator has been implicated in multiple cellular activities or biological processes including tRNA modification, histone modification, DNA demethylation or methylation, tubulin acetylation, and exocytosis. Studies in the model plant *Arabidopsis thaliana* suggest that the structure of Elongator and its functions are highly conserved between plants and yeast. Disruption of the Elongator complex in plants leads to aberrant growth and development, resistance to abiotic stresses, and susceptibility to plant pathogens. The morphological and physiological phenotypes of *Arabidopsis* Elongator mutants are associated with decreased histone acetylation and/or altered DNA methylation. This review summarizes recent findings related to the epigenetic function of Elongator in plant development and responses to abiotic and biotic stresses.

## Introduction

Elongator was first identified as an elongating RNA polymerase II (RNAP II)-associated protein complex in yeast (Otero et al., 1999; Wittschieben et al., 1999), and was later found to be highly conserved in eukaryotes (Versées et al., 2010). This complex consists of six subunits (ELP1–ELP6) with ELP1-3 forming the core subcomplex and ELP4–ELP6 the accessory subcomplex (Li et al., 2001; Winkler et al., 2001). Deletion of any of the six subunits results in almost identical phenotypes, suggesting that all six subunits are required for Elongator’s cellular functions (Krogan and Greenblatt, 2001; Nelissen et al., 2005, 2010; Mehlgarten et al., 2010; Glatt et al., 2012). ELP1 has a nuclear localization sequence essential for Elongator function and WD40 repeats that possibly function together with the WD40-containing subunit ELP2 as scaffolds for complex assembly (Fichtner et al., 2003). ELP3 is the catalytic subunit containing a C-terminal GNAT-type histone acetyltransferase (HAT) domain and an N-terminal iron–sulfur (Fe–S) radical *S*-adenosylmethionine (SAM) domain (Wittschieben et al., 1999; Chinenov, 2002). ELP4, ELP5, and ELP6 each form a RecA-ATPase-like fold and together assemble into a hexameric ring-shaped structure (Glatt et al., 2012; Lin et al., 2012).

The presence of a conserved HAT domain in ELP3 and the co-purification of Elongator with elongating RNAP II led to the initial assumption that Elongator might facilitate transcription elongation via histone acetylation (Otero et al., 1999; Wittschieben et al., 1999). Indeed, ELP3 is capable of acetylating all four core histones *in vitro* (Wittschieben et al., 1999), and highly purified holo-Elongator has a dominant preference for lysine-14 of histone H3 and to a small extent for lysine-8 of Histone H4 (Winkler et al., 2002; Li et al., 2009). Consistently, yeast, human, and plant Elongator mutants contain reduced levels of acetylated histone H3 and H4 (Kim et al., 2002; Winkler et al., 2002; Close et al., 2006; Nelissen et al., 2010). Although chromatin immunoprecipitation (ChIP) failed to detect enrichment of Elongator at actively transcribed genomic regions in yeast (Pokholok et al., 2002), RNA immunoprecipitation (RIP) showed that Elongator interacts with nascent mRNA during transcription elongation (Gilbert et al., 2004). Conversely, in humans and plants, ChIP experiments detected association of Elongator with gene promoters and/or coding regions (Kim et al., 2002; Close et al., 2006; Wang et al., 2013). Furthermore, it was recently reported that the ELP4–ELP6 accessory subcomplex assembles into a hexameric ring-shaped structure that is important for recognizing histone H3 (Lin et al., 2012). These results, together with the finding that Elongator facilitates RNAP II transcription through chromatin in an acetyl-CoA-dependent manner (Kim et al., 2002), support that Elongator assists RNAP II during transcription elongation via chromatin remodeling.

ELP3 also contains a putative SAM-binding domain, which was hypothesized to function catalytically in histone demethylation (Chinenov, 2002). In yeast, the radical SAM domain of ELP3 was shown to be a motif required for the structural integrity of Elongator (Greenwood et al., 2009). In contrast, the archaea *Methanocaldococcus jannaschii* ELP3 SAM-binding motif might have a catalytic role, since it binds and cleaves SAM (Paraskevopoulou et al., 2006). Interestingly, a recent study in mouse indicated that Elongator is required for zygotic paternal genome demethylation, which is mediated by the ELP3 radical SAM domain rather than the HAT domain (Okada et al., 2010).

Accumulating evidence suggests that Elongator also plays a role in formation of the 5-methoxycarbonylmethyl (mcm^5^) and 5-carbamoylmethyl (ncm^5^) side chains on uridines at the wobble position in tRNAs (Karlsborn et al., 2015). Yeast Elongator mutants lack tRNA modifications at wobble uridines or thiouridines at position 34 of the anticodon (Huang et al., 2005). Interestingly, elevated levels of two tRNA species rescue the defects of transcription and exocytosis in yeast Elongator mutants (Esberg et al., 2006), and overexpression of tRNA^Lys^_UUU_ complements the stress-related phenotypes of the yeast *sin3*/*elp3* mutant cells (Fernández-Vázquez et al., 2013). These results are in line with the recent finding that the hexameric ELP456 accessory subcomplex specifically recognizes tRNA (Glatt et al., 2012). Therefore, it was proposed that the effects of *elp* mutations on transcription and secretion might be indirect consequences of inappropriate tRNA modifications (Esberg et al., 2006).

**FIGURE 1:**
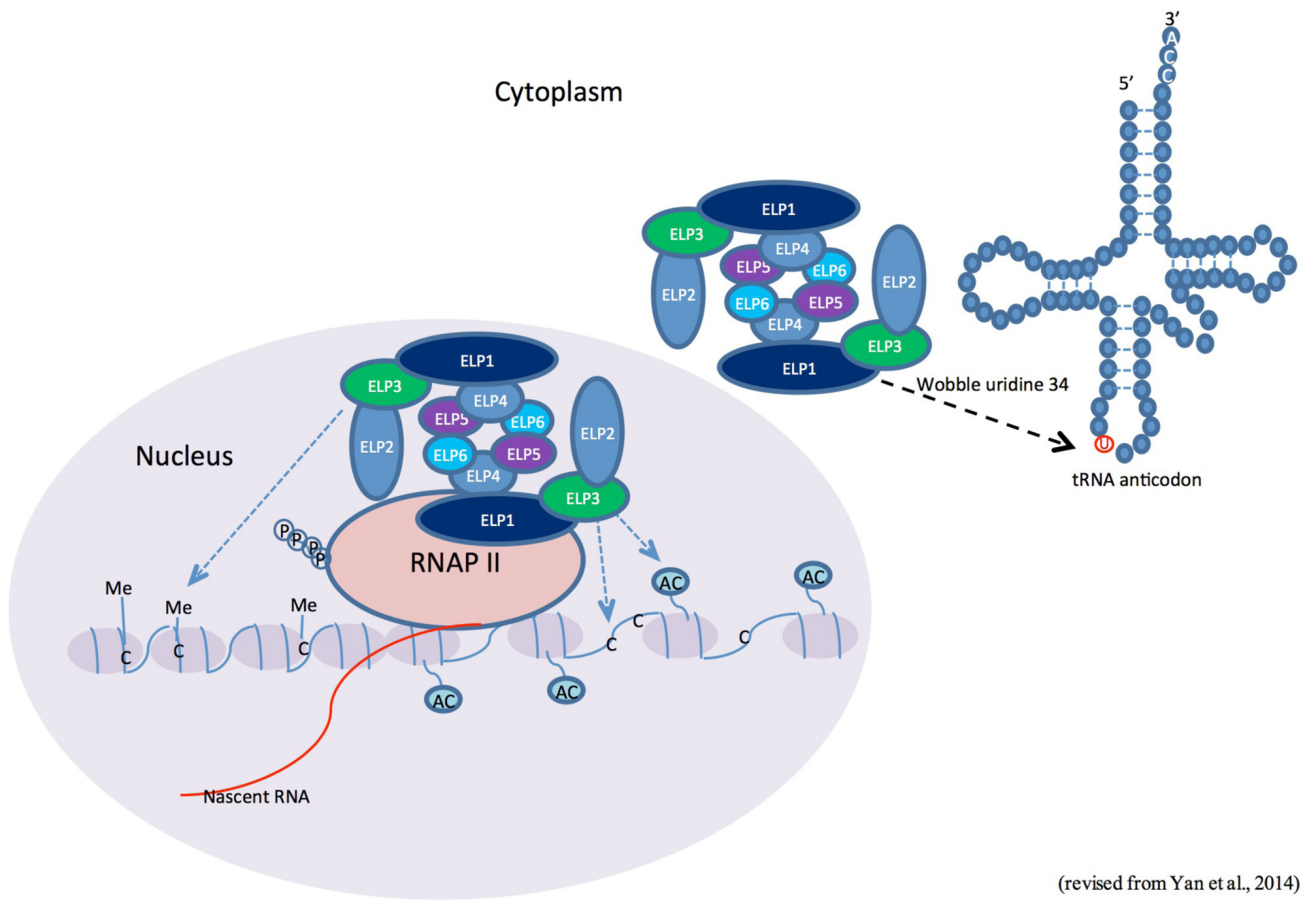
**Possible roles of the Elongator complex in plants.** The currently available information suggests three possible roles for the Elongator complex in plants. In the nucleus, Elongator is associated with hyperphosphorylated RNA polymerase (RNAP II) and is required for histone acetylation and/or DNA demethylation/methylation at various genetic loci. On the other hand, Elongator may also regulate protein translation through tRNA modification in the cytoplasm.

In addition to its functions in histone acetylation and tRNA modification, Elongator has also been implicated in multiple kingdom-specific activities, such as exocytosis in yeast and neuronal development in animals (Rahl et al., 2005; Close et al., 2006). Recent studies performed in the model plant *Arabidopsis thaliana* have revealed that the structure and function of Elongator are conserved in plants (**Figure 1**; **Table 1**; Nelissen et al., 2010; DeFraia and Mou, 2011; Van Lijsebettens et al., 2014; Yan et al., 2014). This review focuses on recent advances in the study of the epigenetic function of Elongator in plant development and responses to biotic and abiotic stresses.

**Table 1:** A timeline for the establishment of Elongator’s function in plants.

Elongator mutant/subunit	Topic	Reference
*elo1*, *elo2*, *elo3*, *elo4*	Mutants with abnormal shaped leaves	Berná et al. (1999)
ELO4/DRL1	Identification of ELO4 as DRL1, a homolog of the yeast Elongator-associated protein KTI12	Nelissen et al. (2003)
ELO1/AtELP4, ELO2/AtELP1, ELO3/ AtELP3	Identification of three Elongator subunits and their function in cell proliferation during organ growth in *Arabidopsis*	Nelissen et al. (2005)
*abo1*/*elo2*/*Atelp1*	Role of ABO1/ELO2/AtELP1 in modulating ABA and drought responses; functional conservation between AtELP1 and yeast ELP1	Chen et al. (2006)
ELO1/AtELP4	Cytological investigation of the *elo1*/*Atelp4* mutant for ELO4/AtELP1’s function in leaf lateral growth	Falcone et al. (2007)
HAG3/AtELP3	Involvement of HAG3/AtELP3 in *Agrobacterium*-mediated root transformation	Crane and Gelvin (2007)
ELO1/AtELP4, ABO1/ELO2/AtELP1, AtELP2, AtELP6	Role of four Elongator subunits in ABA response, oxidative stress, and anthocyanin biosynthesis; different functions of two subcomplexes in ABA-mediated stomatal movement	Zhou et al. (2009)
All six subunits	Purification of the *Arabidopsis* Elongator complex; epigenetic function of ELO3/AtELP3 in auxin signaling	Nelissen et al. (2010)
ELO2/AtELP1, ELO3/AtELP3	Functional conservation of tRNA modifications between plants and yeast	Mehlgarten et al. (2010)
AtELP1, ELO4/DRL1	Role of AtELP1 and ELO4/DRL1 in ncm^5^ uridine modifications of tRNA	Chen et al. (2010)
*gns1*/*Atelp2*	Role of GNS1/AtELP2 in plant immunity	DeFraia et al. (2010)
ELO3/AtELP3	Function of ELO3/AtELP3 in the establishment of leaf adaxial–abaxial polarity	Kojima et al. (2011)
ELO1/AtELP4, ELO2/AtELP1, ELO3/AtELP3, ELO4/DRL1	Function of three Elongator subunits in regulating mitotic cell cycle and leaf patterning in *Arabidopsis*	Xu et al. (2012)
AtELP2	Epigenetic function of AtELP2 in plant immunity	Wang et al. (2013)
*gns2*/*Atelp3*	Role of AtELP3 and its histone acetyltransferase (HAT) and *S*-adenosylmethionine (SAM) domains in plant immunity	DeFraia et al. (2013)
SlELP2L, a tomato Elongator complex protein 2-like protein	Function of SlELP2L in tomato growth and development	Zhu et al. (2015)

## Identification and Characterization of the Elongator Complex in Plants

In a genetic screen for mutants with abnormal shaped leaves in *Arabidopsis*, four *elongata* (*elo*) mutants, *elo1*, *elo2*, *elo3*, and *elo4*, were isolated (Berná et al., 1999). The *elo4* mutant was later found to be allelic to the *drl1-2* (*deformed roots and leaves1*) mutant, which carries a transposed *Ds* element in the *Arabidopsis* homolog of the yeast Elongator-associated protein KTI12 (KILLER TOXIN INSENSITIVE12; Fichtner et al., 2002; Nelissen et al., 2003). Further BLAST search indicated that homologs of all six subunits of the yeast Elongator are also present in the *Arabidopsis* genome (Nelissen et al., 2003). Indeed, the *elo1*, *elo2*, and *elo3* mutations were identified in the *A. thaliana* (*At*) Elongator subunits *AtELP4*, *AtELP1*, and *AtELP3*, respectively, (Nelissen et al., 2005).

To verify the existence and composition of an Elongator complex in plants, tandem affinity purification (TAP) was performed using *Arabidopsis* cell suspension cultures overexpressing TAP-tagged ELO3/AtELP3, ELO1/AtELP4, or AtELP5 (Nelissen et al., 2010). All six subunits (ELO2/AtELP1, AtELP2, ELO3/AtELP3, ELO1/AtELP4, AtELP5, and AtELP6) were purified in the TAPs with ELO3/AtELP3, ELO1/AtELP4, or AtELP5 as bait, confirming that the Elongator composition is conserved in plants. Moreover, stoichiometric concentrations of ELO2/AtELP1, AtELP2, and ELO3/AtELP3 were found on gels when ELO2/AtELP1 was used as bait, whereas only stoichiometric concentrations of ELO1/AtELP4, AtELP5, and AtELP6 were detected when using ELO1/AtELP4 or AtELP5 as bait, suggesting that the *Arabidopsis* holo-Elongator is also composed of two distinct subcomplexes. The structural conservation was further supported by the fact that AtELP6 interacts with both ELO1/AtELP4 and AtELP5 in yeast two-hybrid assays (Nelissen et al., 2010).

ELO3/AtELP3 was shown to colocalize with euchromatin and the phosphorylated form of RNAP II, indicating that plant Elongator is also involved in the process of RNAP II transcription elongation (Nelissen et al., 2010). The functional conservation was further corroborated by results from heterologous complementation experiments. Ectopic expression of *ELO2*/*AtELP1* in a yeast *elp1* mutant restores sensitivity toward zymocin, a yeast killer toxin complex (Chen et al., 2006), and ELO2/AtELP1 and ELO3/AtELP3 are able to structurally replace the respective yeast Elongator subunits and functionally restore ochre suppression and γ-toxin sensitivity of a yeast *elp1 elp3* double mutant by reconstituting U34 tRNA modifications (Mehlgarten et al., 2010). Moreover, tRNA wobble uridine modifications are compromised in the *Arabidopsis elo2*/*Atelp1*, *elo3*/*Atelp3*, and *drl1* mutants (Chen et al., 2010; Mehlgarten et al., 2010). Therefore, the structure of Elongator and its functions in RNAP II transcription elongation as well as tRNA modification are conserved between yeast and plants.

## The Epigenetic Function of Elongator in Plant Growth and Development

As *elo*/*Atelp* mutants exhibit abnormal shaped leaves (Berná et al., 1999; Nelissen et al., 2003, 2005), Elongator clearly plays an important role in plant growth and development. At the macroscopic level, *elo*/*Atelp* mutants have narrow and elongated leaves, reduced primary roots and lateral root density, abnormal inflorescence phyllotaxis, delayed seedling growth, and reduced apical dominance (Nelissen et al., 2003, 2005, 2010). At the cellular and ultrastructural levels, leaves of *elo*/*Atelp* mutants have larger and fewer cells, which show less stacked grana in the chloroplasts, a hypotonic vacuole, and massive presence of Golgi vesicles in the cytoplasm (Falcone et al., 2007).

Since the phytohormone auxin plays a leading role in regulating cell proliferation (Wang and Ruan, 2013), the abnormal phenotypes of *elo*/*Atelp* mutants might be due to defects in auxin signaling or distribution. Indeed, a group of auxin-related genes are down-regulated in *elo*/*Atelp* mutants (Nelissen et al., 2010). Interestingly, decreased expression of two auxin-related genes, *SHY2*/*IAA3* and *LAX2*, in the *elo3*/*Atelp3* mutant is correlated with reduced histone H3K14 acetylation at the coding regions and 3′-UTRs (**Table 2**), suggesting that these two genes might be direct targets for Elongator’s HAT activity during RNAP II transcription elongation. On the other hand, H3K14 acetylation levels in several other auxin-related genes, which are also down-regulated in *elo3*/*Atelp3*, are not changed, indicating that Elongator likely targets specific genes for histone acetylation rather than affecting overall histone acetylation levels (Nelissen et al., 2010). Additionally, ethylene (ET) and jasmonic acid (JA) signaling and abiotic stress responses are up-regulated in *elo*/*Atelp* plants, which might also contribute to their pleiotropic phenotypes (Chen et al., 2006; Zhou et al., 2009; Nelissen et al., 2010).

**Table 2:** Epigenetic changes in *Arabidopsis* Elongator mutants.

Epigenetic change	Target gene/region	Biological process	Reference
Histone acetylation	Histone H3K14 at the coding regions and/or 3’-UTRs of *SHY2*/*IAA3* and *LAX2* in *elo3*	Auxin signaling/cell proliferation	Nelissen et al. (2010)
	Histone H3K9/14 at the coding regions of *NPR1*, *PR2*, *PR5*, *EDS1*, and *PAD4* in *Atelp2*	Salicylic acid (SA) signaling/plant immunity	Wang et al. (2013)
	Cellular levels of acetylated histone H3 and H4 and chromatin-bound H3K56 and H4K5 within replicons in *elo3*	Mitotic cell cycle	Xu et al. (2012)
	Histone H3K9/14 at the coding regions of *WRKY33*, *ORA59*, and *PDF1.2* in *Atelp2*	JA/ET signaling/plant immunity	Wang and Mou (unpublished data)
DNA methylation	DNA methylation landscape and pathogen-induced DNA methylation changes at the *NPR1* promoter region and the *PAD4* coding region in *Atelp2*	SA signaling/plant immunity	Wang et al. (2013)

It is well known that normal cell proliferation is required for the leaf adaxial–abaxial polarity establishment in *Arabidopsis* (Yuan et al., 2010). The abnormal leaf polarity formation in *elo*/*Atelp* mutants is likely due to the defective cell cycle progression caused by aberrant DNA replication and increased DNA damage (Xu et al., 2012). In yeast, Elongator associates with PCNA (proliferating cell nuclear antigen) and functions in maintenance of genome stability (Li et al., 2009). Elongator also interacts with PCNA in *Arabidopsis*, and the interaction is required for DNA replication and repair. Moreover, ELO3/AtELP3 is required for cellular histone H3 and H4 acetylation and DNA replication-coupled H3K56 and H4K5 acetylation (**Table 2**), which are important for DNA replication-coupled chromatin assembly (Xu et al., 2012). Thus, Elongator likely modulates mitotic cell cycle through interacting with PCNA and functioning in histone acetylation.

Elongator may also mediate the establishment of leaf adaxial–abaxial polarity in *Arabidopsis* by repressing transcription of abaxial-determinant genes and class 1 *KNOX* genes (Kojima et al., 2011). This negative role of Elongator in gene expression may be an indirect consequence of positively regulated genes.

Recently, the function of a tomato *AtELP2*-like gene, *SlELP2L*, was characterized (Zhu et al., 2015). Similar to *Atelp* mutants, *SlELP2L*-RNAi transgenic tomato plants display pleiotropic phenotypes, such as delayed seedling development, reduced leaf growth, rapidly senescing leaves and sepals, and dark-green fruits. A number of ET- and ripening-related genes are down-regulated in *SlELP2L*-silencing plants, whereas several DNA methyltransferase genes are up-regulated. It was therefore proposed that the tomato SlELP2L might regulate plant growth and development by modulating DNA methylation. Additionally, levels of GA and IAA, which have profound effects on plant growth and development (Martí et al., 2006; Zhao, 2010), are reduced in *SlELP2L*-RNAi plants. Interestingly, some phenotypes of the *SlELP2L*-RNAi tomato plants are in marked contrast to those of *elo*/*Atelp* mutants. For instance, ET signaling is upregulated in *elo*/*Atelp* mutants (Nelissen et al., 2010), but down-regulated in *SlELP2L*-silencing plants (Zhu et al., 2015). Moreover, *elo*/*Atelp* mutants accumulate high levels of auxin, whereas *SlELP2L*-RNAi tomato plants exhibit reduced levels of auxin. These differences suggest that the function of Elongator in different plant species may not be exactly the same.

## The Role of Elongator in Plant Responses to Abiotic Stresses

In a genetic screen for *Arabidopsis* mutants with altered drought sensitivity, a drought-resistant mutant, *abo1-1* (ABA-overly sensitive), was identified (Chen et al., 2006). The *abo1-1* mutant is hypersensitive to ABA in stomatal closure and seedling growth and carries a mutation in the *ELO2*/*AtELP1* gene. In a separate genetic screen for mutants hypersensitive to ABA in root growth, *Atelp2* and *Atelp6* were identified (Zhou et al., 2009). It was found that *abo1*/*elo2*/*Atelp1*, *Atelp2*, *elo1*/*Atelp4*, and *Atelp6* are all hypersensitive to ABA in seed germination and seedling growth. Similarly, the tomato *SlELP2L*-silencing plants exhibit an obvious increase in ABA sensitivity during seedling growth (Zhu et al., 2015). On the other hand, only the core subcomplex mutants *abo1*/*elo2*/*Atelp1* and *Atelp2*, but not the accessory subcomplex mutants *elo1*/*Atelp4* and *Atelp6*, exhibit ABA hypersensitivity in stomatal closure (Zhou et al., 2009). The different functions of the two subcomplexes in ABA-mediated stomatal movement appear to conflict with the notion that both core and accessory subcomplexes are essential for the function of the holo-Elongator described to date (Svejstrup, 2007; Van Lijsebettens et al., 2014; Karlsborn et al., 2015).

Disruption of the *Arabidopsis* Elongator complex results in increased resistance to the oxidative stress caused by CsCl and methyl viologen under light, indicating that Elongator functions as a negative modulator of oxidative stress (Zhou et al., 2009). Consistently, expression of *CAT3* (*CATALASE 3*), which encodes a catalase decomposing hydrogen peroxide in reactive species homeostasis, is up-regulated in *elo*/*Atelp* mutants. Elevated expression of *CAT3* may contribute to the increased oxidative stress resistance. The *elo*/*Atelp* mutants also accumulate high levels of basal and light-induced anthocyanins (Zhou et al., 2009). Since anthocyanins function as antioxidants to protect plants from oxidative stress caused by diverse stressors such as drought, salt, and light (Gould, 2004), the elevated levels of anthocyanins in *elo*/*Atelp* mutants might also contribute to the enhanced resistance to oxidative stress.

## The Epigenetic Function of Elongator in Plant Responses to Biotic Stresses

Salicylic acid (SA) is a key defense signal molecule against biotrophic and hemibiotrophic pathogens in plants (Vlot et al., 2009). SA accumulation occurs after pathogen infection, which is essential for activation of both local and systemic acquired resistance (SAR; Durrant and Dong, 2004). The transcription coactivator NPR1 [NONEXPRESSOR OF PATHOGENESIS-RELATED (PR) GENES] is a master regulator of SA-mediated defense responses (Durrant and Dong, 2004). Mutations in the *NPR1* gene significantly compromise basal immunity, effector-triggered immunity (ETI), and SAR (Durrant and Dong, 2004). These mutations also lead to SA hyperaccumulation during pathogen infection (Shah et al., 1997; Wildermuth et al., 2001) and failure of seedling development on Murashige and Skoog (MS) medium containing high concentrations of SA (Cao et al., 1997). In a genetic screen for *gns* (*green npr1 seedling on SA medium*) mutants, we found that mutations in *AtELP2* and *ELO3*/*AtELP3* restore SA tolerance to *npr1* on half-strength MS medium containing 0.5 mM SA and also suppress *npr1*-mediated SA hyperaccumulation (DeFraia et al., 2010, 2013). Since high levels of SA trigger production of reactive oxygen species and subsequent cellular damage (Rao et al., 1997), Elongator may facilitate SA toxicity by suppressing the expression of antioxidant genes such as *CAT3* (Zhou et al., 2009).

Mutations in *AtELP2* and *ELO3*/*AtELP3* also compromise plant immunity (DeFraia et al., 2010, 2013). While *gns1*/*Atelp2* and *gns2*/*Atelp3* are more susceptible to both virulent and avirulent *Pseudomonas syringae*, SAR induction in these mutants is normal. Interestingly, simultaneous removal of AtELP2 and NPR1 completely abolishes resistance to two different ETI-inducing pathogens, *P. syringae* pv. *tomato* (*Pst*) DC3000/*avrRpt2* and *Pst* DC3000/*avrRps4*. Microarray analysis revealed that the *gns1*/*Atelp2* mutation has a broader impact than *npr1* on *Pst* DC3000/*avrRpt2*-induced transcriptome changes, indicating that AtELP2 is a more general modulator of transcription than NPR1. Furthermore, *Pst* DC3000/*avrRpt2*-induced expression of a group of SA pathway defense genes is delayed and/or decreased in the *gns1*/*Atelp2* mutant compared to wild type. Similarly, both *gns1*/*Atelp2* and *gns2*/*Atelp3* are significantly more susceptible than wild type to the necrotrophic fungal pathogens *Botrytis cinerea* and *Alternaria brassicicola* and *B. cinerea*-induced expression of the JA/ET pathway defense genes *OCTADECANOID-RESPONSIVE ARABIDOPSIS AP2/ERF59* (*ORA59*) and *PLANT DEFENSIN1.2* (*PDF1.2*) is delayed and/or decreased in *gns1*/*Atelp2* plants (Wang and Mou, unpublished data). These results together suggest that AtELP2 is an accelerator of plant defense responses (DeFraia et al., 2010).

**FIGURE 2:**
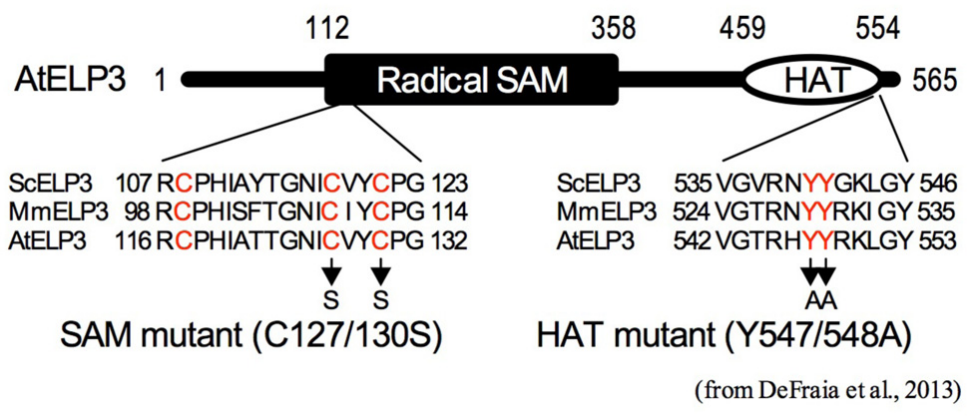
**Schematic representation of the histone acetyltransferase (HAT) and radical *S*-adenosylmethionine (SAM) domains of AtELP3.** The radical SAM and HAT domains of AtELP3 are aligned with those of the yeast (ScELP3) and mouse ELP3 (MmELP3). Only sequences that are part of the alignment are shown. The conserved amino acid residues are labeled in red. Arrows indicate the mutations created in the SAM and HAT mutants (DeFraia et al., 2013).

Genome-wide DNA methylation analysis revealed that the *gns1*/*Atelp2* mutation increases total number of methylcytosines, decreases average methylation levels of methylcytosines, and alters methylation levels of specific cytosines (Wang et al., 2013). Further analysis showed that AtELP2 is required for pathogen-induced dynamic changes in DNA methylation levels of two major defense genes *NPR1* and *PAD4* (**Table 2**). On the other hand, histone acetylation assay indicated that histone H3K9/14ac levels in the coding regions of several defense genes, including *NPR1*, *PAD4*, *EDS1*, *PR2*, *PR5*, *WRK33*, *ORA59*, and *PDF1.2*, are significantly reduced in *gns1*/*Atelp2* (**Table 2**). The reduced histone H3K9/14ac levels are correlated with delayed and/or decreased induction of these defense genes in *gns1*/*Atelp2*, suggesting a role for AtELP2 in histone acetylation (Wang et al., 2013; Wang and Mou, unpublished data). Consistently, mutants of the *Arabidopsis* Elongator catalytic subunit (ELO3/AtELP3) lacking the conserved residues in either the HAT domain or the radical SAM domain (**Figure 2**), fail to complement *gns2*/*Atelp3* mutant phenotypes (DeFraia et al., 2013), indicating that both domains are required for Elongator to function in *Arabidopsis*. Therefore, Elongator likely plays an epigenetic role in response to pathogen infections.

## Conclusions and Perspectives

Studies in *Arabidopsis* indicate that the structure of the Elongator complex and its functions are highly conserved between plants and yeast (Otero et al., 1999; Winkler et al., 2002; Nelissen et al., 2010). Disruption of the Elongator complex in *Arabidopsis* leads to pleiotropic growth and defense phenotypes, which could be attributed to delayed and/or decreased expression of some genes involved in these processes (Nelissen et al., 2005; Zhou et al., 2009; DeFraia et al., 2010; Xu et al., 2012). The delayed and/or decreased gene expression in *Atelp* mutants is associated with reduced histone acetylation and/or altered DNA methylation (Nelissen et al., 2010; Wang et al., 2013). Although the intrinsic relationship between histone acetylation and DNA demethylation/methylation in *Arabidopsis* Elongator mutants remains to be determined, these results suggest that Elongator may epigenetically modulate plant development and responses to abiotic and biotic stresses.

However, a direct role of Elongator in histone acetylation and DNA demethylation or methylation in plants has not been established. Moreover, the enzymatic activities (HAT and DNA demethylase or methyltransferase) of the catalytic subunit ELP3 in plants have not been tested. Without such information, it would be difficult to fully appreciate the epigenetic function of Elongator in plants. Additionally, recent evidence suggests that herbivore and pathogen attack of plants generates defense phenotypes across generations and such transgenerational memory appears to be associated with DNA methylation, histone modifications, and small RNAs (Holeski et al., 2012; Luna et al., 2012; Rasmann et al., 2012; Slaughter et al., 2012). Since Elongator modulates histone acetylation and DNA methylation (Nelissen et al., 2010; Xu et al., 2012; Wang et al., 2013), it would be interesting to test whether Elongator is also involved in transgenerational defense induction and epigenetic inheritance in plants.

Accumulating evidence suggests that the yeast Elongator may primarily function in tRNA modification (Karlsborn et al., 2015). Studies in *Arabidopsis* also indicate that Elongator may play a role in tRNA modification in plants (Chen et al., 2010; Mehlgarten et al., 2010). However, the connection between Elongator’s function in tRNA modification and plant development and responses to abiotic and biotic stresses still remains elusive. It would be very interesting to test whether overexpression of certain types of hypomodified tRNAs (tRNA^Lys^_UUU_, tRNA^Gln^_UUG_, and tRNA^Glu^_UUC_) could rescue some of the plant Elongator mutant phenotypes. Such experiments would help resolve the mystery of the multitasking role of Elongator in plants.

## Author Contributions

YD and ZM wrote the manuscript.

## Conflict of Interest Statement

The authors declare that the research was conducted in the absence of any commercial or financial relationships that could be construed as a potential conflict of interest.
